# A Multi-Input Neural Network for Microwave Hemorrhagic Stroke Identification Using Multimodal Data

**DOI:** 10.3390/brainsci16030274

**Published:** 2026-02-28

**Authors:** Zekun Zhang, Heng Liu, Ruide Li, Huiyuan Zhu, Fan Li, Xianchao Zhang, Yao Zhai

**Affiliations:** 1 School of Information and Electronics, Beijing Institute of Technology, Beijing 100081, China; 2 School of Cyberspace Science and Technology, Beijing Institute of Technology, Beijing 100081, China; 3 Yangtze Delta Region Academy of Beijing Institute of Technology, Jiaxing 314019, China; 4 Provincial Key Laboratory of Multimodal Perceiving and Intelligent Systems, Jiaxing University, Jiaxing 314001, China; 5 Department of Nursing, College of Medicine, Jiaxing University, Jiaxing 314001, China

**Keywords:** deep learning, hemorrhagic stroke identification, multimodal fusion, microwave imaging, temporal encoder

## Abstract

**Background:** Hemorrhagic stroke is a life-threatening cerebrovascular disease, and early identification is crucial for timely clinical intervention. Microwave imaging is non-ionizing, portable, and low-cost, and thus has potential for pre-hospital and bedside screening; however, existing methods often suffer from limited reconstruction resolution, scarce data, and suboptimal information utilization when only a single modality is used. **Methods:** We propose a dual-channel, multi-input multimodal deep neural network for hemorrhagic stroke recognition, which jointly exploits complementary features from microwave images and time-domain waveforms and performs feature-level cross-modal fusion. A high-fidelity microwave brain simulation dataset is constructed for model training, and multiple temporal encoding strategies are systematically evaluated. **Results:** The proposed multimodal model achieves improved accuracy and stability compared with single-modality baselines and conventional approaches, demonstrating the benefit of cross-modal feature fusion for microwave-based hemorrhage recognition. **Conclusions:** Multimodal learning can enhance discrimination and robustness in microwave-based hemorrhage recognition, supporting its potential use for rapid, non-ionizing pre-hospital and bedside assessment.

## 1. Introduction

Stroke is one of the leading causes of death and long-term disability worldwide [1]. Hemorrhagic stroke accounts for only 10–20% of all stroke cases but leads to disproportionately high mortality and morbidity [2]. Because this subtype often progresses rapidly, early diagnosis and timely intervention are crucial, yet accurately identifying the location and extent of intracranial hemorrhage in the hyperacute stage remains challenging [3,4]. Current neuroimaging for hemorrhage assessment relies mainly on computed tomography (CT) [5] and magnetic resonance imaging (MRI) [6]. CT offers high sensitivity but uses ionizing radiation and bulky hardware, limiting pre-hospital deployment [7], whereas MRI avoids radiation but is time-consuming, costly, and operationally complex [8]. These limitations have motivated the development of compact, non-ionizing, and portable imaging modalities for real-time brain monitoring in pre-hospital and bedside settings [9].

Among the candidate techniques, microwave imaging (MWI) has attracted growing interest as a low-cost, non-ionizing approach that exploits dielectric contrast between healthy and pathological tissues [10,11,12]. In typical brain MWI systems, a multi-antenna array is deployed around the head, microwave signals propagate through brain tissues, and the resulting scattered fields are acquired at multiple ports [13,14,15,16]. Through inverse reconstruction or beamforming, spatial energy maps can be produced to indicate potential lesions [12]. Numerous phantom experiments and prototype systems have verified the technical feasibility of this approach for hemorrhagic lesion detection [17,18]. However, in realistic scenarios the high dielectric constant and strong absorption of the skull introduce severe aberrations and energy distortion [19], while amplitude–phase imbalance among channels, calibration errors, and individual anatomical variations further generate noise and artifacts [20]. Consequently, reconstructed microwave images often suffer from limited resolution and unstable quality, and robust recognition under limited and noisy measurements remains an open challenge.

Algorithmically, existing MWI pipelines primarily emphasize image formation, whereas downstream lesion recognition under limited and imperfect measurements is less directly addressed. Nonlinear inverse approaches such as the Born iterative method (BIM) [21,22] and contrast source inversion (CSI) [23,24] can improve quantitative accuracy but are computationally demanding and sensitive to model mismatch. Linearized methods such as truncated singular value decomposition (TSVD) improve stability at the cost of spatial detail [25,26]. Confocal microwave imaging (CMI) provides a correlation-based beamforming alternative that avoids large-scale optimization and enables rapid energy-map generation [27,28]. Adaptive CMI (ACMI) further incorporates numerically computed background field distributions into the imaging kernel to improve focusing and reduce artifacts. Despite these advances, learning-based recognition remains challenged by limited measurements and imperfect physics, and existing pipelines still underutilize the raw signal domain and lack unified, physics-informed evaluation of temporal modeling and fusion strategies [29,30].

Beyond simulations and phantoms, early patient studies have reported encouraging clinical feasibility. Persson et al. conducted proof-of-principle clinical measurements using a wearable microwave helmet and a data-driven classifier with CT reference [20]. Abbosh et al. reported results from a portable electromagnetic (microwave-band) brain scanner evaluated on 50 stroke patients, showing high classification accuracy and coarse 2-D localization capability [31]. Feasibility studies have also investigated workflow integration of point-of-care microwave stroke devices in emergency departments [32]. However, available patient datasets remain small, heterogeneous, and often site- or device-dependent, which hinders systematic benchmarking and data-hungry model training [33]. Consequently, many deep-learning studies rely on electromagnetic simulations to generate scalable, well-labeled datasets for controlled evaluation [34,35]. Guo et al. proposed a feature-space learning-based inversion framework for 3-D stroke imaging and trained the model using simulation-built head databases to learn structural priors [36], while real measurements are frequently used for feasibility assessment or perturbation characterization rather than large-scale training [37]. In addition, microwave data representations are tightly coupled to acquisition configurations, including antenna geometry, operating band or pulse design, coupling-medium properties, and calibration protocols, which further limits direct reuse across platforms.

These constraints motivate a physics-informed, quantitatively controlled multimodal framework that provides paired signal–image data and enables fair ablations of temporal encoders and fusion strategies. In practice, microwave measurements can be represented as time-domain waveforms or frequency-domain scattering parameters, which are mutually transformable via FFT and both encode amplitude and phase information. Time-domain waveforms can be efficiently generated in large quantities by finite-element-method (FEM) simulations and are suitable for controlled training after truncation and preprocessing [38]. In parallel, ACMI and related algorithms transform signals into interpretable spatial maps that emphasize lesion localization. Therefore, waveforms and images provide complementary views of the same scattering process, but most existing learning-based studies still treat them in isolation or use ad hoc fusion designs.

Although multimodal fusion is a natural way to exploit this complementarity, current multimodal approaches for microwave stroke imaging remain fragmented. Prior work has often used shallow feature concatenation or coarse decision-level fusion, without a unified design and systematic comparison of temporal encoders—such as one-dimensional convolution [39], dilated convolution [40], temporal convolutional networks (TCNs) [41], long short-term memory (LSTM) networks [42], and Transformers [43]—under the same dataset and fusion architecture [44]. Moreover, heterogeneous modalities differ in sampling rate, dynamic range, and noise statistics, posing challenges for cross-modal alignment and robust fusion [45,46]. From an engineering perspective, there is still a lack of a physics-informed, quantitatively controlled experimental framework that (i) provides paired waveform–image data tailored for deep models and (ii) clarifies how temporal encoder design impacts multimodal fusion performance and robustness under realistic variability [33,46].

Motivated by these gaps, this paper proposes a physics-informed multimodal learning framework for hemorrhagic stroke recognition based on microwave data. We build a high-fidelity simulation pipeline that couples FEM-generated time-domain waveforms with field-distribution-enhanced ACMI images, and design a dual-channel multi-input neural network that performs feature-level fusion of these two modalities for hemorrhage classification. Within this unified framework, we further conduct a comprehensive ablation study of temporal encoders to clarify how different time-series architectures affect multimodal performance under controlled noise and variability.

The main contributions of this work are summarized as follows:

1. Physics-informed multimodal dataset. We construct a high-fidelity microwave brain simulation dataset that jointly provides time-domain waveforms and field-distribution-enhanced confocal images. The pipeline incorporates anatomical variability, skull-induced distortion, and system noise, thereby generating paired waveform–image data suitable for controlled training and ablation studies.
2. Dual-channel multi-input architecture for waveform–image fusion. We develop a dual-channel neural network that combines a ResNet-based image encoder with a 1D CNN–LSTM waveform encoder and performs feature-level fusion. By leveraging the complementarity between spatially localized microwave images and temporally informative waveforms, the proposed architecture achieves higher accuracy and improved robustness for hemorrhagic stroke identification than single-modality models and conventional MWI pipelines.
3. Unified evaluation of temporal encoders in multimodal MWI. We establish a unified experimental framework that systematically compares multiple time-series encoders for microwave waveforms, including 1D CNN–LSTM, temporal convolutional networks (TCNs), dilated CNNs, and Transformer-based variants. The resulting ablation study clarifies the impact of temporal modeling choices on multimodal fusion performance and provides engineering guidance under the investigated simulation conditions.

The remainder of this paper is organized as follows. Section 2 describes the construction of the multimodal dataset, including FEM-based simulation of time-domain waveforms, signal preprocessing, and generation of confocal imaging results. Section 3 presents the architecture of the proposed dual-channel multi-input neural network, detailing the ResNet-based image branch, the 1D CNN–LSTM waveform branch, and the feature fusion strategy. Section 4 reports the performance evaluation and ablation experiments on different temporal encoders and modality configurations. Section 5 discusses the implications for multimodal fusion and device design in practical stroke triage. Section 6 concludes the paper and outlines directions for future research.

## 2. Dataset Acquisition and Preprocessing

In this study, a deep learning model is developed to identify brain hemorrhage from microwave sensing data, including raw time-domain waveforms and reconstructed confocal images. Because real-patient microwave neuroimaging data remain limited and are difficult to standardize across institutions and acquisition hardware, a high-fidelity FEM-based simulation framework is adopted to generate controlled and diverse datasets. The simulations incorporate anatomical variability, propagation-induced distortion, and additive noise, while preserving spatial and temporal scattering characteristics. This section describes the FEM simulation setup, the confocal image reconstruction procedure, and the preprocessing pipeline.

At the current stage, large-scale real-patient microwave stroke measurements are difficult to acquire and harmonize due to ethical and regulatory constraints, limited device availability, and the need for reliable labels aligned with CT or MRI. Therefore, a simulation-based dataset is used to enable controlled and reproducible evaluation of temporal encoders and multimodal fusion under consistent acquisition settings.

### 2.1. Time-Domain Simulation Setup

A time-domain electromagnetic model was constructed using the finite element method to simulate wave propagation within the human head. A heterogeneous cranial phantom was placed at the center of a circular antenna array comprising 12 identical elements, uniformly positioned on a circle of radius 10 cm. The space between the phantom and the antenna array was filled with a homogeneous coupling medium with a relative permittivity of $\varepsilon_{r}=45$ and conductivity σ=0.5S/m, to ensure good wave penetration and impedance matching.

**Figure 1:**
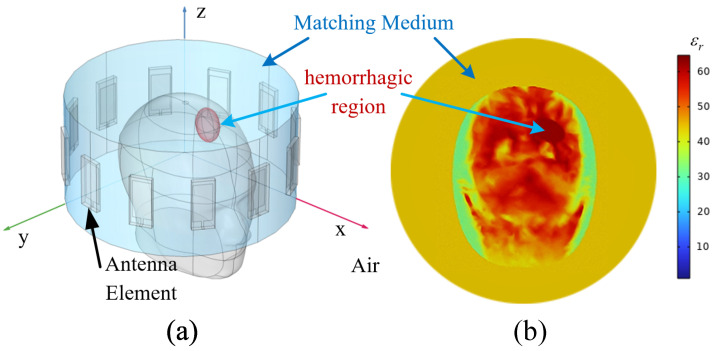
Simulation model configuration for FEM-based time-domain microwave sensing. (**a**) Geometric layout of the 12-element circular antenna array with a radius of 10 cm surrounding the head phantom. The coupling medium is shown in light blue and fills the gap between the array and the phantom. Representative hemorrhagic inclusions are shown as red ellipses. (**b**) Cross-sectional map of the relative permittivity on the array plane. It illustrates the heterogeneous dielectric distribution used for wave propagation and subsequent image formation.

The overall simulation configuration is illustrated in Figure 1. Panel (a) shows the geometric layout, including the circular antenna array, the coupling medium (light blue), and representative hemorrhagic regions (red ellipses). Panel (b) presents a cross-sectional view of the relative permittivity distribution on the antenna plane. Each antenna transmitted a differentiated Gaussian pulse defined by:

(1) $$s\left(t_{n}\right)=\left[1-4\pi {\left(\frac{t_{n}-t_{0}}{\tau }\right)}^{2}\right]\cdot e^{-2\pi {\left(\frac{t_{n}-t_{0}}{\tau }\right)}^{2}},$$

with τ=0.4 ns and *t*_0_ = 1.2 ns. The simulation was conducted with a time step of 0.03 ns and a total duration of 9 ns, resulting in 300-time samples for each waveform. In each simulation run, the antennas were excited sequentially, with one antenna acting as the transmitter and the remaining antennas acting as receivers, producing a full time-domain signal matrix $S\in R^{12\times 12\times 300}$, where the diagonal entries are zero vectors and self-transmission was excluded. Each element $S_{i,j}$ denotes the time-domain waveform received at antenna *j* when antenna *i* transmits.

Three classes of brain conditions were simulated:

1. Normal brain without lesions.
2. Brain with one localized hemorrhagic region.
3. Brain with two spatially separated hemorrhages.

To enhance variability, each instance of the head model and hemorrhage configuration was randomly rotated, deformed, and scaled, and additive Gaussian noise was introduced to achieve a signal-to-noise ratio (SNR) of 5 dB.

### 2.2. Adaptive Confocal Microwave Imaging

**Figure 2:**
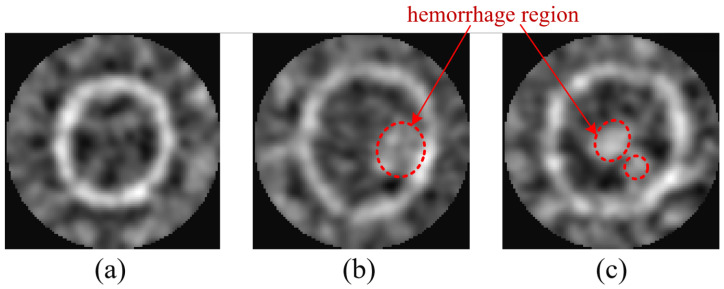
Representative adaptive confocal microwave images used in the dataset. (**a**) Normal brain without lesions. (**b**) Brain with a single hemorrhagic region. (**c**) Brain with two spatially separated hemorrhages. Red circles represent the hemorrhage locations in the imaging plane.

The full wave solver was three-dimensional while imaging was carried out on the array plane $\Omega =\{\left(x,y,z_{0}\right)\}$. A background forward simulation without lesions was performed in the same geometry and materials to characterize the transmit to pixel propagation on Ω. As shown in Figure 2, the microwave brain image dataset covers three representative categories corresponding to normal, single-hemorrhage, and double-hemorrhage cases. To spatially localize the scattering response, an adaptive confocal microwave imaging method was applied to the time-domain data. The imaging procedure exploited the knowledge of the incident electric field distribution to enhance focus and suppress background noise.

For each transmitter *i* and pixel $r=(x,y,z_{0})\in \Omega$, let $e_{i}\left(r,t_{n}\right)$ denote complex time-domain electric field at r generated by the source waveform $s(t_{n})$. The source spectrum is defined as $S\left(\omega \right)=F\{s\left(t_{n}\right)\}$, and $E_{i}\left(r,\omega \right)=F\left\{e_{i}\left(r,t_{n}\right)\right\}$ is subsequently used in constructing the imaging kernel. The background single-path transfer function from transmitter *i* to r is estimated in the frequency domain as:

(2) $$H_{i}\left(r,\omega \right)=\frac{E_{i}\left(r,\omega \right)}{S(\omega )+\gamma },$$

where γ is a small Tikhonov stabilizer that prevents numerical blow-up near spectral notches of S(ω). The two-way model assisted kernel is then defined by:

(3) $$K_{ij}\left(r,\omega \right)=S\left(\omega \right)H_{i}\left(r,\omega \right)H_{j}\left(r,\omega \right),$$

which contains the source spectrum and preserves the three-dimensional background propagation i→r and r→j.

The experimental data are processed in a differential and whitened fashion. Let $x_{ij}\left(t_{n}\right)$ and $x_{ij}^{\mathrm{bg}}\left(t_{n}\right)$ denote the recorded waveforms with and without the head respectively. The differential signal is computed as $\Delta x_{ij}\left(t_{n}\right)=x_{ij}\left(t_{n}\right)-x_{ij}^{\mathrm{bg}}\left(t_{n}\right)$, which suppresses static system and interface responses. The whitened matched image *I* is computed as:

(4) $$I\left(r\right)=\sum_{i=1}^{12}\sum_{j=1}^{12}\sum_{\omega }\frac{\Delta X_{ij}\left(\omega \right)K_{ij}^{*}\left(r,\omega \right)}{N_{ij}\left(\omega \right)+\epsilon },$$

where ${\left(\cdot \right)}^{*}$ denotes complex conjugation and the sum over ω is restricted to the effective antenna bandwidth, $\Delta X_{ij}\left(\omega \right)$ is the discrete Fourier transform of $\Delta x_{ij}\left(t_{n}\right)$, $N_{ij}\left(\omega \right)$ is the noise power spectral density estimated from background recordings, and ϵ is added to provide numeric robustness. The time domain counterpart follows from inverse transforms and corresponds to correlating the whitened differential data with the time reversed kernel $k_{ij}\left(r,t_{n}\right)=\left(s*h_{i}\left(r,\cdot \right)*h_{j}\left(r,\cdot \right)\right)\left(t_{n}\right)$, where $h_{i}$ is the impulse response associated with $H_{i}$.

The image domain Ω was discretized into a 100×100 square pixel grid covering the circular field of view enclosed by the antenna array. The final grayscale image reflects the spatial energy distribution and serves as a structural input to the classification model.

### 2.3. Time-Domain and Multimodal Data Preprocessing

To reduce the impact of multipath interference and emphasize primary reflections, the original time-domain tensor $S\in R^{12\times 12\times 300}$ is truncated to retain only the first 64-time samples after the primary arrival. The truncated tensor $S_{\mathrm{trunc}}\in R^{12\times 12\times 64}$ reduces the influence of late-time multipath and reflection noise and contains essential scattering information.

The final dataset consists of a total of 4808 labeled samples, including 2000 samples without hemorrhage, 2000 samples with a single hemorrhagic lesion, and 808 samples with two hemorrhagic lesions, covering a range of anatomical and scattering variations. For each sample, two data modalities are provided: (i) a two-dimensional grayscale confocal image; and (ii) a raw time-domain waveform matrix. These two modalities are paired one-to-one and labeled according to their respective diagnostic category.

To adapt the waveform data for convolutional processing, the 3D tensor is reshaped into a two-dimensional matrix of size 64×144, where the 144 columns correspond to the flattened transmitter-receiver channels. This configuration allows 1D convolutional kernels to extract temporal features across all spatial channels simultaneously.

In parallel, the confocal images are used as spatial priors and are paired with the waveform matrices for multimodal learning. The test set remains unaltered to ensure fair and reproducible evaluation.

Importantly, no additional network-side normalization is applied to either modality. This does not mean that the inputs are “raw and unscaled” in a strict sense. In the simulation pipeline, all samples are generated using the same excitation pulse and consistent material and boundary settings, so the time-domain responses already lie on a common amplitude scale across the dataset. Applying further per-sample normalization could suppress physically meaningful amplitude cues that reflect scattering strength and loss.

This choice is also consistent with practical microwave measurement workflows. In vector network analysis, S-parameters are defined from incident and received wave quantities and represent amplitude and phase ratios, and calibration is routinely performed to improve measurement accuracy and correct systematic errors [47]. In addition, differential or background-referenced processing is widely used in microwave imaging to suppress static responses and emphasize target-induced variations by subtracting a baseline or background measurement [11,48]. In the present study, confocal images are generated under the same reconstruction pipeline and therefore exhibit stable intensity ranges without pronounced scale shifts.

## 3. Proposed Method

### 3.1. Multi-Input Network Architecture

**Figure 3:**
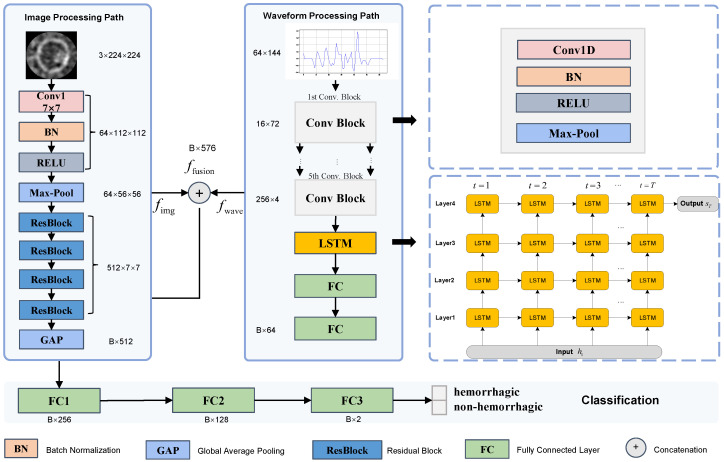
Overall architecture of the proposed multimodal neural network for hemorrhagic stroke classification. The framework consists of an image processing path based on ResNet and a waveform processing path using 1D CNN and LSTM, followed by feature fusion and final classification.

To better identify hemorrhagic lesions in microwave brain images, this paper proposes a multi-input neural network architecture based on a dual-branch structure. The proposed method employs dual-channel feature extraction and multimodal fusion strategies, jointly learning spatial features from microwave brain images and temporal features from time-domain waveforms, in order to enhance the discriminative ability between normal and abnormal samples. The architecture mainly consists of three core modules: an image feature extraction branch, a time-domain waveform feature extraction branch, and a multimodal feature fusion and classification module. The overall structure is illustrated in Figure 3.

#### 3.1.1. Image Processing Path

The image branch is built upon the ResNet-18 architecture, utilizing a pretrained ResNet-18 model as the backbone to extract spatial features from simulated microwave brain images. Specifically, the first 17 convolutional layers are retained to preserve the hierarchical residual characteristics of the network. The architecture includes an initial 7×7 convolutional layer, followed by a 3×3 max-pooling layer, and four residual stages, each comprising two residual units. The number of channels in each stage is 64, 128, 256, and 512, respectively. A global average pooling layer at the end produces a 512-dimensional image feature vector. To enhance training stability and mitigate overfitting, skip connections are employed to add the input features to the output at the end of the module, thereby alleviating the vanishing gradient problem.

Let the *X* be the input feature map derived from the initial image *I*, and the convolutional kernel weights as *W*, the residual block can be formulated as:

(5) $$Y=\mathrm{ReLU}(BN\left(W_{2}^{\mathrm{img}}*\mathrm{ReLU}\left(BN\left(W_{1}^{\mathrm{img}}*X\right)\right)\right)+X).$$

Here, $W_{1}^{\mathrm{img}}$ and $W_{2}^{\mathrm{img}}$ denote the convolutional kernel weights, ∗ represents the two-dimensional convolution operation, and *X* is the input feature map. BN(·) denotes the batch normalization operation, ReLU(·) refers to the Rectified Linear Unit activation function, and *Y* is the output feature map of the residual block.

A global average pooling (GAP) operation is applied after the final group of convolutional layers to compress the multi-channel feature map into a 512-dimensional vector:

(6) $$f_{\mathrm{img}}=\mathrm{GAP}\left(X_{\mathrm{Res}}\right),$$

where $X_{\mathrm{Res}}$ denotes the feature map output by ResNet, and $f_{\mathrm{img}}$ is the resulting image feature vector.

#### 3.1.2. Waveform Processing Path

The waveform branch receives the raw time-domain signals and processes them into a 64×144 two-dimensional matrix, from which dynamic features are extracted using a five-stage 1D convolutional module followed by a stacked LSTM structure. Specifically, the 1D convolutional module consists of five convolutional layers, each utilizing a one-dimensional convolution with kernel size 5, along with batch normalization, ReLU activation, and max pooling. Max pooling is performed with a stride of 2 for downsampling. The *i*-th convolution operation is defined as:

(7) $$c_{i}=\mathrm{MaxPool}\left(\mathrm{ReLU}\left(\mathrm{BN}\left(W_{i}^{\mathrm{wave}}*c_{i-1}+b_{i}^{\mathrm{wave}}\right)\right)\right),$$

where $c_{0}$ denotes the input waveform data, and $W_{i}^{\mathrm{wave}}$ and $b_{i}^{\mathrm{wave}}$ represent the weights and biases of the *i*-th convolutional layer, respectively.

The number of channels across the five convolutional layers increases sequentially as 16→32→64→128→256. The final output tensor has a shape of [256,4], representing high-level local temporal features across four time steps. A four-layer stacked unidirectional LSTM network follows the convolutional module to capture nonlinear temporal dependencies across time steps. Let the input at time step *t* be $h_{t}\in R^{256}$, which denotes the feature vector at the *i*-th time step of the pooled waveform representation. Then the hidden and cell states of the LSTM are updated as:

(8) $$\left(s_{t},c_{t}\right)=\mathrm{LSTM}\left(h_{t},s_{t-1},c_{t-1}\right),$$

where $s_{t}$ and $c_{t}$ are the hidden and cell states at time *t*, and $s_{t-1},c_{t-1}$ are the states from the previous step.

The final hidden state $s_{T}\in R^{64}$ is retained as the global feature representation of the waveform. This is further processed by two fully connected layers for dimensionality reduction:

(9) $$f_{\mathrm{wave}}=FC_{2}\left(\mathrm{ReLU}\left(FC_{1}\left(s_{T}\right)\right)\right),$$

where $f_{\mathrm{wave}}\in R^{64}$ is the final waveform feature vector.

#### 3.1.3. Feature Fusion and Binary Classification

The 512-dimensional feature vector from the image branch and the 64-dimensional vector from the waveform branch are concatenated to form a unified 576-dimensional feature vector:

(10) $$f_{\mathrm{fusion}}=\left[f_{\mathrm{img}};f_{\mathrm{wave}}\right],$$

where [·;·] denotes the vector concatenation operation, and $f_{\mathrm{fusion}}\in R^{576}$. This fused feature is then passed through a three-layer fully connected classifier:

(11) $$z_{1}=\mathrm{Dropout}\left(\mathrm{ReLU}\left(W_{1}^{\mathrm{fusion}}f_{\mathrm{fusion}}+b_{1}^{\mathrm{fusion}}\right)\right),$$

(12) $$z_{2}=\mathrm{ReLU}\left(W_{2}^{\mathrm{fusion}}z_{1}+b_{2}^{\mathrm{fusion}}\right),$$

(13) $$\hat{y}=\mathrm{Softmax}\left(W_{3}^{\mathrm{fusion}}z_{2}+b_{3}^{\mathrm{fusion}}\right).$$

Here, $W_{i}^{\mathrm{fusion}}$ and $b_{i}^{\mathrm{fusion}}$ represent the weight matrix and bias term of the *i*-th layer (i=1,2,3), respectively, and the dropout rate is set to 0.5 to mitigate overfitting. The final output $\hat{y}\in R^{2}$ denotes the probability distribution indicating whether the input image belongs to the category of hemorrhagic stroke or non-hemorrhagic class.

### 3.2. Loss Function

To enhance the model’s identification capability in the binary classification task of microwave brain images, particularly for hemorrhagic stroke samples, this work employs a weighted cross-entropy loss function. Specifically, the identification task is formulated as a binary classification problem, where normal samples without hematoma are labeled as class 0, and abnormal samples with hematoma that include both single and double hematoma cases are uniformly labeled as class 1.

Although hemorrhagic samples slightly outnumber normal samples in the dataset, a weighted cross-entropy loss is used during training to emphasize the model’s focus on the high-risk abnormal class, thereby reducing the risks of misclassification and missed diagnosis. By assigning different weights to each class, the loss function improves the model’s ability to discriminate the abnormal category.

In this study, a weight of $w_{1}=5.0$ is assigned to hemorrhagic (abnormal) samples and a weight of $w_{0}=1.0$ is assigned to normal samples. The mathematical formulation of the weighted cross-entropy loss is defined as:

(14) $$L=-\frac{1}{N}\sum_{i=1}^{N}\left(w_{1}y_{i}\log\left(p_{i}\right)+w_{0}\left(1-y_{i}\right)\log\left(1-p_{i}\right)\right),$$

where *N* denotes the number of samples, $y_{i}\in \left\{0,1\right\}$ is the ground-truth label of the *i*-th sample, and $p_{i}$ represents the predicted probability of being abnormal. The class weight was selected based on validation performance with an emphasis on reducing false negatives for the abnormal class.

### 3.3. Implementation Details

All experiments in this study were conducted on a computing platform equipped with an Intel Core i9-12900KF processor, 32 GB of memory, and an NVIDIA GeForce RTX 4090 graphics processing unit, with CUDA used for acceleration. The model was implemented using the PyTorch (v2.4.0) deep learning framework. In the data preprocessing stage, the spatial resolution of the microwave brain images was resized from the original 100×100 to 224×224 pixels to meet the input requirements of the network. The original size of the time-domain waveform data was 12×12×300. The waveform tensor is truncated to the first 64 samples after the primary arrival and reshaped into a 64×144 matrix by vectorizing the transmitter–receiver channels. Self-transmission channels correspond to zero vectors and are kept as zeros for a fixed input shape.

For dataset splitting, stratified sampling is used based on the original three categories (normal, single hematoma, and double hematoma). The dataset is divided into a training set (80%) and a test set (20%), preserving the class proportions in both sets. From the training set, 10% of samples are further split as a validation set using stratified sampling for early stopping and model selection. Each sample is generated with independently randomized parameters, including lesion geometry and location and an independent noise realization, which reduces near-duplicate samples. Notably, the test set is held out exclusively for final evaluation and is not used for training, model selection, or hyperparameter tuning.

As shown in Table 1, The hyperparameters for model training were set as follows: the number of training epochs was 50, and the batch size was 16. The Adam optimizer was used with an initial learning rate of $1\times 10^{-4}$, which was kept constant during training. The weight decay was set to $1\times 10^{-4}$ to reduce overfitting. The gradient clipping threshold was set to 1.0 to prevent gradient explosion. A dropout rate of 0.5 was applied to the fully connected layers and part of the convolutional layers to improve generalization ability. The loss function for the classification task was weighted cross-entropy to address the class imbalance problem, with a higher weight assigned to the abnormal class. The performance on the validation set was used as the reference for early stopping to avoid overfitting.

## 4. Performance Evaluation and Experimental Results

This section aims to systematically evaluate the performance of the proposed method on the binary classification task, namely distinguishing between normal and abnormal microwave brain images with hematomas. We employed a series of widely recognized evaluation metrics for quantitative analysis and reported the experimental results in detail from the perspectives of classification accuracy and model robustness, in order to fully validate the effectiveness of the proposed method.

### 4.1. Evaluation Metrics

To evaluate classification performance, this study reports Accuracy, Recall, Precision, Area Under the Receiver Operating Characteristic Curve (ROC-AUC), Area Under the Precision–Recall Curve (PRC-AUC), and F1-Score. For each metric, a 95% confidence interval is estimated using a non-parametric bootstrap resampling method with 1000 iterations, providing uncertainty quantification beyond point estimates and supporting robust performance assessment.

#### 4.1.1. Accuracy

Accuracy intuitively indicates the overall capability of the model to distinguish between normal and abnormal brain microwave images.

(15) $$Accuracy=\frac{TP+TN}{TP+TN+FP+FN}.$$

where TP (True Positives) refers to the number of images correctly predicted as abnormal (hemorrhagic); TN (True Negatives) refers to the number of images correctly predicted as normal; FP (False Positives) denotes the number of normal images incorrectly predicted as abnormal; and FN (False Negatives) denotes the number of abnormal images incorrectly predicted as normal.

#### 4.1.2. Recall

Recall measures the proportion of abnormal images that are correctly identified. In medical diagnostic scenarios, a high recall rate can significantly reduce the risk of missed diagnoses, which is crucial for the timely detection of hemorrhagic stroke cases.

(16) $$Recall=\frac{TP}{TP+FN}.$$

#### 4.1.3. Precision

Higher precision means that images predicted as abnormal are more likely to be truly abnormal, effectively reducing false alarms and enhancing the credibility of the diagnosis.

(17) $$Precision=\frac{TP}{TP+FP}.$$

#### 4.1.4. Area Under the ROC Curve (ROC-AUC)

ROC-AUC represents the area under this curve. Ideally, a perfect classifier has a ROC-AUC value of 1, while a random classifier has a value of 0.5. In this study, ROC-AUC is used to comprehensively evaluate the model’s ability to distinguish between normal and abnormal images across all possible thresholds, fully reflecting the model’s discriminative performance.

#### 4.1.5. Area Under the Precision-Recall Curve (PRC-AUC)

Given the typically low prevalence of positive samples in medical imaging, PRC-AUC quantifies the area under the precision-recall curve, effectively evaluating the trade-off between recall and precision at various thresholds, thereby providing a critical reference for model performance evaluation.

#### 4.1.6. F1-Score

The F1-Score, as the harmonic mean of precision and recall, assigns equal importance to both precision and recall, offering a balanced evaluation metric.

(18) $$F1-Score=2\times \frac{\mathrm{Precision}\times \mathrm{Recall}}{\mathrm{Precision}+\mathrm{Recall}}.$$

Bootstrap-based 95% confidence intervals quantify metric variability due to test-set resampling and provide a robust estimate of performance uncertainty, enabling reliable evaluation of the proposed model for hemorrhagic stroke recognition from microwave images.

### 4.2. Classification Results

In this section, the classification performance of the proposed method on the multimodal dataset was evaluated and compared with multiple baseline methods. The evaluation metrics include Accuracy, Recall, Precision, F1-score, ROC-AUC, and PRC-AUC, with the mean values and their 95% confidence intervals reported to ensure the reliability of the statistical results.

#### 4.2.1. Overall Performance

Table 2 summarizes the classification results of the proposed method and baseline approaches on multimodal, unimodal image, and unimodal time-domain data. The proposed method consistently outperforms all alternatives across metrics, with concentrated confidence intervals that indicate stable model performance.

**Figure 4:**
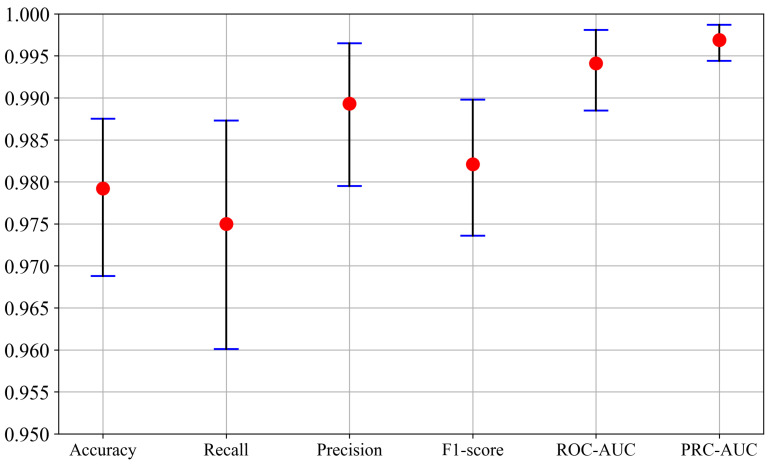
Overall performance of the proposed multimodal network across six evaluation metrics on the test set. Red markers show mean values and error bars denote the 95% confidence intervals, indicating performance stability across evaluations.

As illustrated in Figure 4, specifically, the proposed method demonstrated strong performance across all evaluation metrics on the test set:

- Accuracy reached 97.92% (95% CI: 96.88–98.75).
- Recall was 97.50% (95% CI: 96.01–98.73).
- Precision hit 98.93% (95% CI: 97.95–99.65).
- F1-score attained 98.21% (95% CI: 97.36–98.98).
- ROC-AUC reached 0.9941 (95% CI: 0.9885–0.9981).
- PRC-AUC achieved 0.9969 (95% CI: 0.9944–0.9987).

**Figure 5:**
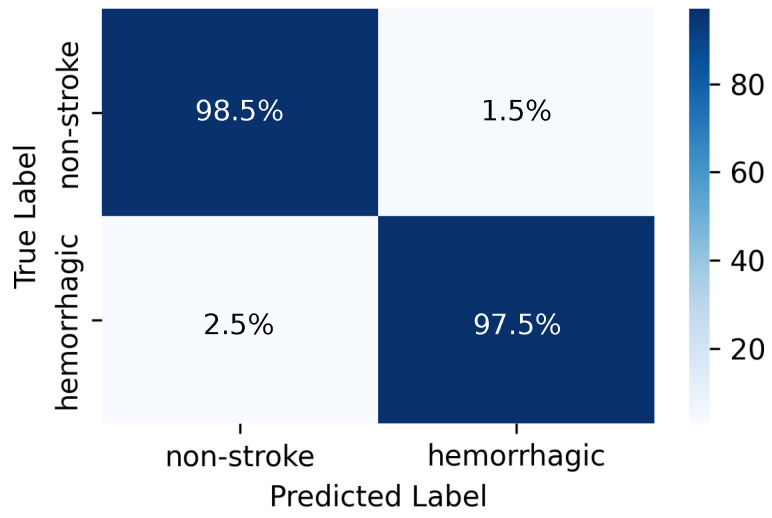
Confusion matrix of the proposed method for classifying hemorrhagic stroke and normal cases. Diagonal cells represent correct predictions and off-diagonal cells correspond to misclassifications. The model achieves a recall of 98.50% for non-stroke and 97.50% for hemorrhagic stroke.

Figure 5 shows high class-wise recall, with 98.50% for normal samples and 97.50% for hemorrhagic samples. The narrow confidence intervals across metrics reflect consistent discriminative capability under different thresholds.

The task is formulated as a binary classification of ‘normal vs. abnormal (including single and double hematomas)’. High recall reduces missed detections, while high precision limits false alarms. ROC-AUC and PRC-AUC values near 1 confirm strong discrimination of abnormal samples under varying thresholds, highlighting the advantage of multimodal fusion.

Overall, the method delivers high accuracy and stable discrimination between positive and negative classes. For screening-oriented settings, high recall helps reduce missed abnormal cases, while high precision limits false alarms. The near-unity AUC values indicate strong discrimination under the evaluated acquisition configuration. Although the confocal images are generated from the multistatic measurements, they provide a structured spatial representation that is not equivalent to the raw waveforms and can emphasize different cues. The unimodal baselines, particularly waveform-only models, remain clearly inferior, suggesting that performance is not driven by a single trivial feature and that fusion leverages complementary information. Moreover, the test set is strictly held out and used only for final evaluation, reducing the likelihood of inflated performance due to data reuse.

#### 4.2.2. Statistical Significance of Multimodal Improvement

To test whether multimodal fusion yields statistically significant improvements over unimodal baselines, paired bootstrap resampling is performed on the held-out test set. For each bootstrap replicate, the same resampled indices are used to evaluate the proposed model and a baseline model, and the metric difference Δm is computed for each metric *m*. A two-sided bootstrap *p*-value is estimated as p=2min{Pr(Δm≤0),Pr(Δm≥0)}, and the 95% confidence interval of Δm is reported. Table 3 summarizes the resulting confidence intervals and *p*-values for key metrics.

#### 4.2.3. Comparison of Multimodal Methods

On the dataset used in this study, three additional multimodal models were evaluated, namely ResNet + CNN + Transformer, ResNet + WaveformDilatedCNN, and ResNet + TCN + LSTM.

**Figure 6:**
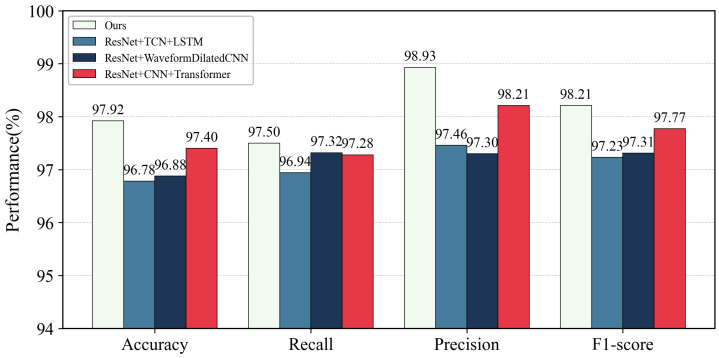
Performance comparison among multimodal fusion models on the test set, including the proposed method and three alternatives (ResNet + CNN + Transformer, ResNet + WaveformDilatedCNN, and ResNet + TCN + LSTM). Bars report the mean Accuracy, Recall, Precision, and F1-score for each method, highlighting the relative benefits of different temporal encoders under the same image backbone and feature-level fusion setting.

The proposed method achieved the best performance across all metrics. As shown in Figure 6, its Accuracy of 97.92% exceeds the 97.40% of ResNet + CNN + Transformer by 0.52%, and is about 1.1 percentage points higher than ResNet + TCN + LSTM (96.78%) and ResNet + WaveformDilatedCNN (96.88%). For F1-score, the proposed method reached 98.21%, 0.44% higher than the 97.77% of ResNet + CNN + Transformer. Precision (98.93%) and Recall (97.50%) also surpassed all comparison models. These consistent improvements within a near-saturated multimodal setting highlight the effectiveness of the proposed feature fusion and temporal encoding strategy.

For threshold-related metrics, the proposed method achieved ROC-AUC of 0.9941 and PRC-AUC of 0.9969, both clearly higher than alternative models. ResNet + CNN + Transformer produced ROC-AUC of 0.9741 and PRC-AUC of 0.9716, while even the closest model, ResNet + TCN + LSTM (ROC-AUC 0.9931, PRC-AUC 0.9962), remained slightly inferior. These results indicate that under varying decision thresholds, the proposed method exhibits slower performance degradation and better stability.

#### 4.2.4. Comparison of Unimodal Methods

The unimodal results clearly demonstrate the necessity of multimodal fusion.

**Figure 7:**
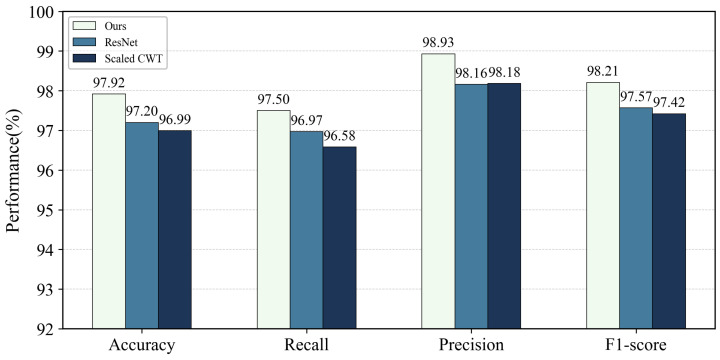
Performance comparison between the proposed multimodal method and unimodal image-based baselines (ResNet and Scaled CWT + 2D CNN). Bars report the mean Accuracy, Recall, Precision, and F1-score, illustrating the performance gap between image-only recognition and waveform—image fusion.

For the image branch, ResNet alone achieved an Accuracy of 97.20%, Recall of 96.97%, F1-score of 97.57%, and ROC-AUC and PRC-AUC of 0.9725 and 0.9700. As shown in Figure 7, these are lower than the proposed method by 0.72 percentage points in Accuracy, 0.64% in F1-score, and by 0.0216 and 0.0269 in ROC-AUC and PRC-AUC. We also compared with the Scaled CWT + 2-D CNN image unimodal method in [37], which achieved 96.99% Accuracy and 97.42% F1-score. Both image unimodal methods showed lower Accuracy (by 0.93%) and F1-score (by 0.79%), with ROC-AUC and PRC-AUC reductions of 0.0234 and 0.0285.

**Figure 8:**
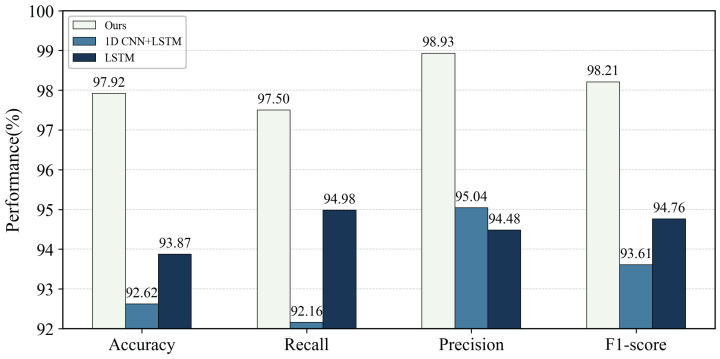
Performance comparison between the proposed multimodal method and unimodal time-domain baselines (1D CNN + LSTM and LSTM). Bars report the mean Accuracy, Recall, Precision, and F1-score, showing the limitation of using temporal signals alone and the advantage of incorporating spatial image cues via multimodal fusion.

The performance gap was more evident in the temporal unimodal branch. As shown in Figure 8, the 1D CNN + LSTM method achieved 92.62% Accuracy, 93.61% F1-score, and ROC-AUC and PRC-AUC of 0.9265 and 0.9214. Compared with the proposed method, Accuracy and F1-score were lower by 5.3% and 4.6%, with ROC-AUC and PRC-AUC reduced by 0.0676 and 0.0755. The pure LSTM method achieved 93.87% Accuracy, 94.98% Recall, 94.48% Precision, and 94.76% F1-score, remaining clearly inferior to both the multimodal and image unimodal approaches.

#### 4.2.5. Ablation Experiment Analysis

To evaluate the contribution of each component in the proposed multimodal fusion model, ablation and substitution experiments were conducted under identical datasets and training settings. The results are shown in Table 4 and Table 5.

Table 4 fixed the image encoder and fusion strategy while varying the temporal encoder. The temporal design had a direct and notable influence on fusion performance. The proposed 1D CNN + LSTM achieved the best results, with 97.92% Accuracy and 98.21% F1-score. Replacing it with other architectures led to clear degradation: TCN + LSTM reduced F1-score to 97.23%, WaveformDilatedCNN to 97.31%, and CNN + Transformer to 97.77%. Pure dilated convolution or self-attention alone did not match the proposed encoder’s effectiveness.

Table 5 further verified the necessity of dual-modality input. Removing any modality caused significant drops in performance. Using only the image branch resulted in 97.20% Accuracy and 97.57% F1-score, while using only the temporal branch reduced them to 92.62% and 93.61%, respectively. Although the image modality provides strong discrimination, temporal waveform data contributes complementary information, and combining both modalities yields superior overall performance. The full model also demonstrated the strongest threshold robustness, outperforming all ablation variants in ROC-AUC (0.9941) and PRC-AUC (0.9969). This indicates that the fusion model maintains more stable decision boundaries across varying thresholds, an important feature in medical scenarios where sensitivity or specificity often needs adjustment.

## 5. Discussion

This study evaluated a dual-branch multimodal fusion framework for hemorrhagic stroke classification using a high-fidelity FEM-based microwave dataset. The results show that integrating spatial image data with temporal waveform data substantially improves performance, offering higher accuracy and robustness than single-modality methods. The proposed architecture combines a ResNet image encoder with a 1D CNN-LSTM temporal encoder, enabling effective extraction and fusion of complementary features. This section further discusses the value of multimodal fusion, the importance of temporal encoder design and its relationship to model robustness, and the clinical implications and future research directions. While the simulation incorporates controlled anatomical variability and additive noise, it does not fully capture all statistical properties and hardware-dependent artifacts of real microwave measurements.

### 5.1. The Value of Multimodal Fusion

The proposed dual-branch framework highlights that microwave waveform signals and reconstructed microwave images provide complementary information for hemorrhage recognition. Spatial images emphasize localization and morphology-related cues, such as energy concentration patterns and boundary contrast, whereas time-domain waveforms preserve transient signatures and phase-consistent dynamics that can be attenuated or distorted during image formation. When either modality is used alone, the model relies on a partial representation of the underlying scattering process, which can reduce stability under noise, channel imbalance, and subject-dependent variability.

Feature-level fusion offers an effective mechanism to combine these complementary cues. Quantitatively, the multimodal model achieved an F1-score of 98.21%, outperforming the image-only (97.57%) and waveform-only (94.76%) variants, indicating that the fused representation better balances sensitivity and specificity. Consistent with this, the image-only branch exhibited reduced threshold stability, with ROC-AUC decreasing by 0.0216–0.0285 compared with the fused model, while the waveform-only branch showed a notable accuracy drop (5.30%) and a characteristic trade-off of high recall but lower precision due to missing spatial context. Ablation experiments further confirmed that removing either modality led to a clear performance degradation, underscoring that both spatial and temporal cues contribute non-redundant information.

From an engineering viewpoint, this design is attractive because predictions can be supported by interpretable image evidence while still leveraging the sensitivity of raw measurements. Overall, these results suggest that multimodal fusion provides a practical balance between interpretability and discriminative power in a screening-oriented workflow, improving robustness across operating thresholds relative to single-modality pipelines.

### 5.2. Time-Domain Encoders and Model Robustness

Temporal encoder design plays a central role in determining how effectively waveform information can be exploited and integrated with image features. The 1D CNN–LSTM encoder combines two complementary capabilities: (i) 1D convolutions capture local pulse structures and abrupt changes that often correlate with scattering events, and (ii) LSTM units model longer-range dependencies and temporal context that may reflect propagation, multi-path effects, and subtle waveform distortions. This hybrid structure provides a good match to microwave time-series characteristics while keeping the model computationally feasible.

The ablation results suggest that alternative sequence models may not yield consistent gains under the investigated data characteristics and scale. For example, purely convolutional temporal models can be limited in capturing long-range dependencies, whereas attention-based models may be more sensitive to data scale, noise statistics, and hyperparameter choices. These observations imply that, for practical implementations, temporal encoders should be selected not only by peak performance but also by stability, efficiency, and ease of calibration across devices.

### 5.3. Clinical Implications and Future Directions

From a system perspective, the observed performance gains indicate that multimodal fusion can be a promising strategy for improving robustness in portable microwave-based screening pipelines, where measurements are often affected by coupling variability, channel mismatch, and limited effective sampling. A fusion model that leverages both raw signals and interpretable images may better tolerate such imperfections than methods that rely on a single representation. In practice, measurement statistics may deviate from the simulated setting due to broader SNR ranges, non-Gaussian interference, and device-specific drift, which motivates further validation on real-world acquisitions.

To support practical deployment, several directions are particularly important. First, pathological coverage should be expanded to reflect realistic case distributions, including more diverse hemorrhage patterns and additional clinically relevant categories such as ischemic stroke and mixed lesions. Second, the fusion mechanism can be strengthened with adaptive or uncertainty-aware weighting so that the network can down-weight unreliable modalities or channels when measurement quality degrades. Third, robustness to device- and site-related variability should be systematically evaluated, including calibration drift, antenna placement perturbations, coupling-medium variation, and inter-subject anatomical differences. Finally, interpretability and reliability measures, such as calibrated confidence estimation and failure detection, will be valuable to facilitate safe integration into time-critical decision support workflows.

## 6. Conclusions

This paper presents a dual-branch multi-input neural network for hemorrhagic stroke recognition using microwave data. The framework integrates field-distribution-enhanced microwave images and time-domain waveforms, where a ResNet-based image encoder extracts spatial features and a 1D CNN–LSTM waveform encoder captures both local pulse patterns and long-range temporal characteristics. Feature-level fusion of the two modalities improves classification performance and robustness compared with single-modality baselines and alternative temporal encoders in our experiments. Collectively, these results support the potential of multimodal microwave learning as a low-cost, non-ionizing screening aid, and help enable faster pre-hospital or bedside risk stratification when integrated into a portable acquisition pipeline.

This study is limited by the scope of the evaluated conditions, including restricted pathological diversity and a specific acquisition configuration, and broader validation is required to establish generalization across hardware and clinical settings. In particular, the current simulations use a fixed SNR setting and simplified noise assumptions, and the modeled anatomical variability may not span the full heterogeneity observed across patients and devices.

In future work, a clinical prototype will be developed to match the simulation configuration in antenna geometry, operating frequency, coupling medium, and calibration protocol, enabling consistent data acquisition and a more faithful assessment of model generalization. Prospective clinical measurements and external validation will then be conducted across subjects and sites, and domain adaptation, calibration-aware fusion, and uncertainty estimation will be further investigated to improve reliability for practical deployment. In addition, microwave imaging results will be compared with standard diagnostic modalities such as CT and MRI to support clinically interpretable validation.

## Figures and Tables

**Table 1:** Hyperparameter Settings for Model Training.

Hyperparameter	Value
Epochs	50
Batch size	16
Optimizer	Adam
Initial learning rate	0.0001
Learning rate schedule	Constant
Weight decay	0.0001
Gradient clipping threshold	1.0
Dropout rate	0.5

**Table 2:** Performance Comparison of Different Methods (Mean and 95% CI).

Modality	Method	Accuracy (%)	Recall (%)	Precision (%)	F1-score (%)	ROC-AUC	PRC-AUC
Multimodal	**The proposed method**	**97.92** **[96.88–98.75]**	**97.50** **[96.01–98.73]**	**98.93** **[97.95–99.65]**	**98.21** **[97.36–98.98]**	**0.9941** **[0.9885–0.9981]**	**0.9969** **[0.9944–0.9987]**
	ResNet + TCN + LSTM	96.78 [95.63–97.82]	96.94 [95.44–98.26]	97.46 [96.09–98.62]	97.23 [96.30–98.14]	0.9931 [0.9874–0.9974]	0.9962 [0.9936–0.9984]
	ResNet + WaveformDilatedCNN	96.88 [95.74–97.82]	97.32 [95.99–98.54]	97.30 [95.88–98.56]	97.31 [96.32–98.24]	0.9683 [0.9565–0.9792]	0.9628 [0.9474–0.9762]
	ResNet + CNN + Transformer	97.40 [96.46–98.44]	97.28 [95.99–98.45]	98.21 [97.13–99.24]	97.77 [96.84–98.62]	0.9741 [0.9635–0.9839]	0.9716 [0.9587–0.9824]
Image Only	ResNet	97.20 [96.15–98.23]	96.97 [95.52–98.29]	98.16 [96.99–99.23]	97.57 [96.64–98.44]	0.9725 [0.9610–0.9818]	0.9700 [0.9561–0.9825]
	Scaled CWT	96.99 [95.95–98.02]	96.58 [95.03–98.02]	98.18 [97.06–99.13]	97.42 [96.53–98.30]	0.9707 [0.9600–0.9816]	0.9684 [0.9542–0.9806]
Time Series Only	1D CNN + LSTM	92.62 [90.85–94.18]	92.16 [89.98–94.14]	95.04 [93.22–96.83]	93.61 [92.08–94.95]	0.9265 [0.9100–0.9421]	0.9214 [0.9020–0.9405]
	LSTM	93.87 [92.31–95.22]	94.98 [93.16–96.70]	94.48 [92.48–96.30]	94.76 [93.32–96.06]	0.9365 [0.9203–0.9524]	0.9268 [0.9064–0.9454]

Note: **Boldface** indicates the best performance among all methods for the corresponding metric.

**Table 3:** Statistical significance of multimodal gains.

Comparison	ΔF1 (pp, 95% CI, *p*)	ΔROC-AUC (95% CI, *p*)	ΔPRC-AUC (95% CI, *p*)
Proposed vs. ResNet (image-only)	0.64(0.15,1.13),p<0.001	0.0216(0.015,0.028),p<0.001	0.0269(0.018,0.036),p<0.001
Proposed vs. 1D CNN + LSTM (waveform-only)	4.60(3.5,5.7),p<0.001	0.0676(0.050,0.085),p<0.001	0.0755(0.058,0.093),p<0.001

Note: Gains are evaluated using paired bootstrap resampling on the test set. Δ denotes the metric difference between the proposed multimodal model and the corresponding baseline.

**Table 4:** Ablation Study of Time-Series Encoder Architectures.

Time-Series Encoder Variant	Accuracy (%)	Recall (%)	Precision (%)	F1-Score (%)	ROC-AUC	PRC-AUC
**1D CNN + LSTM (The proposed method)**	**97.92**	**97.50**	**98.93**	**98.21**	**0.9941**	**0.9969**
TCN + LSTM	96.78	96.94	97.46	97.23	0.9931	0.9962
WaveformDilatedCNN	96.88	97.32	97.30	97.31	0.9683	0.9628
CNN + Transformer	97.40	97.28	98.21	97.77	0.9741	0.9716

Note: All models use ResNet for image encoding and share the same fusion scheme. **Boldface** indicates the best performance among all methods for the corresponding metric.

**Table 5:** Performance comparison of different method combinations.

Method Combination			Performance			
ResNet	1D CNN + LSTM	LSTM	Accuracy	Recall	Precision	F1-Score
✓	×	×	97.20%	96.97%	98.16%	97.57%
×	✓	×	92.62%	92.16%	95.04%	93.61%
×	×	✓	93.87%	94.98%	94.48%	94.76%
✓	✓	✓	97.92%	97.50%	98.93%	98.21%

## Data Availability

Data are available at reasonable request from the corresponding author.The data are not publicly available due to ongoing project agreements and restrictions on sharing intermediate simulation models and code.
